# Detection of Human Papillomavirus in Urogenital Swabs from Male Patients in Northern Serbia

**DOI:** 10.3390/pathogens14060558

**Published:** 2025-06-03

**Authors:** Gordana Kovačević, Vladimir Vuković, Nataša Nikolić, Branka Bašica, Jelena Radovanov, Aleksandra Čolović Popadić, Milica Pejaković Budinski, Tihomir Dugandžija, Zoran Golušin, Aleksandra Patić

**Affiliations:** 1 Institute of Public Health of Vojvodina, 21000 Novi Sad, Serbia; gordana.kovacevic@izjzv.org.rs (G.K.); vladimir.vukovic@mf.uns.ac.rs (V.V.); natasa.nikolic@mf.uns.ac.rs (N.N.); branka.basica@izjzv.org.rs (B.B.); jelena.radovanov@izjzv.org.rs (J.R.); aleksandra.colovic@izjzv.org.rs (A.Č.P.); milica.pejakovic-budinski@mf.uns.ac.rs (M.P.B.); 2 Faculty of Medicine, University of Novi Sad, 21000 Novi Sad, Serbia; zoran.golusin@kcv.rs; 3 Department of Epidemiology, Faculty of Medicine, University of Novi Sad, 21000 Novi Sad, Serbia; dugandzija.tihomir@onk.ns.ac.rs; 4 Department of Microbiology with Parasitology and Immunology, Faculty of Medicine, University of Novi Sad, 21000 Novi Sad, Serbia; 5 Oncology Institute of Vojvodina, 21204 Sremska Kamenica, Serbia; 6 Clinical Centre of Vojvodina, Clinic of Dermatovenereology Diseases, 21000 Novi Sad, Serbia

**Keywords:** human papillomavirus, men, high-risk HPV, low-risk HPV, vaccination

## Abstract

Human papillomavirus (HPV) is one of the most common sexually transmitted infections, affecting both men and women. However, in Serbia, the previous epidemiological and clinical research on HPV has primarily focused on women due to its established role in cervical cancer, while the data on HPV prevalence in men remain scarce. This study analyzed 634 samples from men (mean age: 30 years, SD = 9.98; range: 18–79) from between 2012 and 2024. Overall, 30.76% of the tested men were HPV–positive, with the highest percentage of cases being observed in those aged 25–29 years (32.82%). The most common genotypes were HPV 16 and HPV 31 (22.05% each), followed by HPV6 (20.51%), HPV 56, and HPV 52 (9.23% each). The prevalence of HPV was the highest in the samples from men with genital warts (40.77%). Among the available vaccines, the nonavalent Gardasil 9 provides the broadest protection, covering genotypes found in 82% of the HPV–positive cases identified in this study. Our findings underscore the importance of comprehensive HPV prevention and control measures for the male population, contributing to ongoing research efforts aimed at reducing the burden of HPV–associated diseases in our region. Increasing the vaccination coverage among Serbian men could substantially reduce the overall burden of HPV–related diseases in both sexes.

## 1. Introduction

Human papillomavirus (HPV) infection of the genital tract is globally recognized as one of the most prevalent sexually transmitted infections in both men and women [1]. Despite its common occurrence among sexually active individuals, the epidemiological and clinical investigations of this infection have predominantly focused on women, largely due to HPV’s significant role in cervical cancer etiology. Conversely, research on HPV in males has been comparatively limited, primarily centering on their role in transmitting HPV to women and its contribution to the burden of cervical cancer [2,3].

However, recent data indicate that men not only transmit HPV to their partners but also face significant health implications themselves [4,5,6]. In addition to its well-established role in male anogenital and oropharyngeal cancers, HPV infection has also been investigated as a potential factor in male infertility [7]. HPV DNA has been detected in semen, and evidence suggests that the virus may impair the motility, viability, and morphology of sperm, potentially reducing fertility. Moreover, persistent HPV infection in men has been associated with increased sperm DNA fragmentation, which could negatively impact fertilization and embryo development [7,8]. Beyond its implications for fertility, HPV contributes to the development of certain cancers, such as penile, anal, and head and neck cancer, which exhibit a multifactorial etiology that involves HPV infection [9,10]. The global incidence rates of these carcinomas among men vary by region. Anal cancer, while relatively uncommon, has been increasing in incidence, particularly among men who have sex with men and individuals with human immunodeficiency virus (HIV) [5]. In 2018, the global age-standardized incidence of penile cancer was estimated at 0.80 per 100,000 person-years, and this rate is projected to rise by over 56% by 2040, based on predictions from the GLOBOCAN Cancer Tomorrow tool [11]. Oropharyngeal cancers, particularly those associated with HPV, have been rising in frequency, especially in developed countries [6]. Despite these health implications, the Centers for Disease Control and Prevention (CDC) does not recommend routine HPV testing in men [12]. This recommendation is primarily based on the relatively lower burden of HPV-related health issues in men compared to women. For example, in 2019, HPV caused approximately 620,000 cancer cases in women and 70,000 in men [4]. Routine HPV screening for asymptomatic men is not generally advised due to the absence of standardized guidelines, limited clinical benefit, and the lack of an FDA-approved test or specific treatment for HPV infection [13,14]. Instead, the CDC emphasizes HPV prevention through vaccination, which is the best way to prevent HPV infection and its associated health problems in both men and women, and which can thereby reduce the overall burden of HPV-related diseases in the population [15,16].

The HPV vaccine has been in use since 2006 and was registered in the Republic of Serbia in the same year. The quadrivalent HPV vaccine was initially introduced to the private market, but immunization coverage remained low, particularly among males. In June 2022, the nonavalent HPV vaccine (Gardasil 9) replaced the quadrivalent version and it is now provided free of charge by the state. Although HPV vaccination is not part of the mandatory national immunization program, it is available free of charge to children and teenagers of both genders aged 9–19 years old, and will ideally be administered before the start of sexual activity [17]. Despite these efforts, the vaccination rates among boys remain significantly lower than those among girls [18].

Building upon this context, the present study was designed to assess the baseline frequency of HPV infection in a male population prior to widespread immunization. Given the rarity of male HPV testing in Serbia, this study also aimed to elucidate age-related variations in HPV infection and review the medical conditions prompting men to undergo HPV testing. To the best of our knowledge, this study represents the first investigation of the presence of HPV among men residing in the Autonomous Province of Vojvodina, the northern part of Serbia. The findings of this study can provide critical insights into the epidemiology and clinical aspects of HPV-related diseases, thereby contributing to the advancement of scientific understanding, the enhancement of prevention strategies, and the formulation of effective public health interventions.

## 2. Materials and Methods

### 2.1. Study Design

This retrospective investigation spanned the timeframe from January 2012 to December 2024, and included 634 male participants aged from 18 to 79 years old. The samples for this research were collected in two different ways. The Centre for Virology at the Institute of Public Health of Vojvodina (IPHV), which specializes in routine HPV infection diagnosis, collected 426 urogenital swabs during the study period. Patients were often referred to our center by dermatovenerologists, urologists, and general practitioners.

Additionally, from October 2017 to December 2019, a project titled “I Have Insight into My HPV Profile” was implemented in cooperation with the Institute for Health Protection of Students, Novi Sad. The 250 male students at the University of Novi Sad were encouraged to gain insight into their HPV status to promote their health, sexual education, and the HPV vaccine. A dermatovenerologist examined the participants in this group, who were either asymptomatic or presented with visible warts.

Symptomatic men were defined as those referred for HPV testing due to the presence of clinical signs or symptoms, as documented by the referring physicians. Based on their underlying diagnoses, symptomatic participants were categorized into three subgroups: (1) those with genital warts, (2) those with penile inflammation, and (3) those with urinary infections. Asymptomatic men, by contrast, reported no clinical symptoms at the time of testing. They were tested either because they were considered to be at increased risk of sexually transmitted infections (e.g., partners of HPV-positive women) when being screened as candidates in assisted reproduction programs.

**Figure 1:**
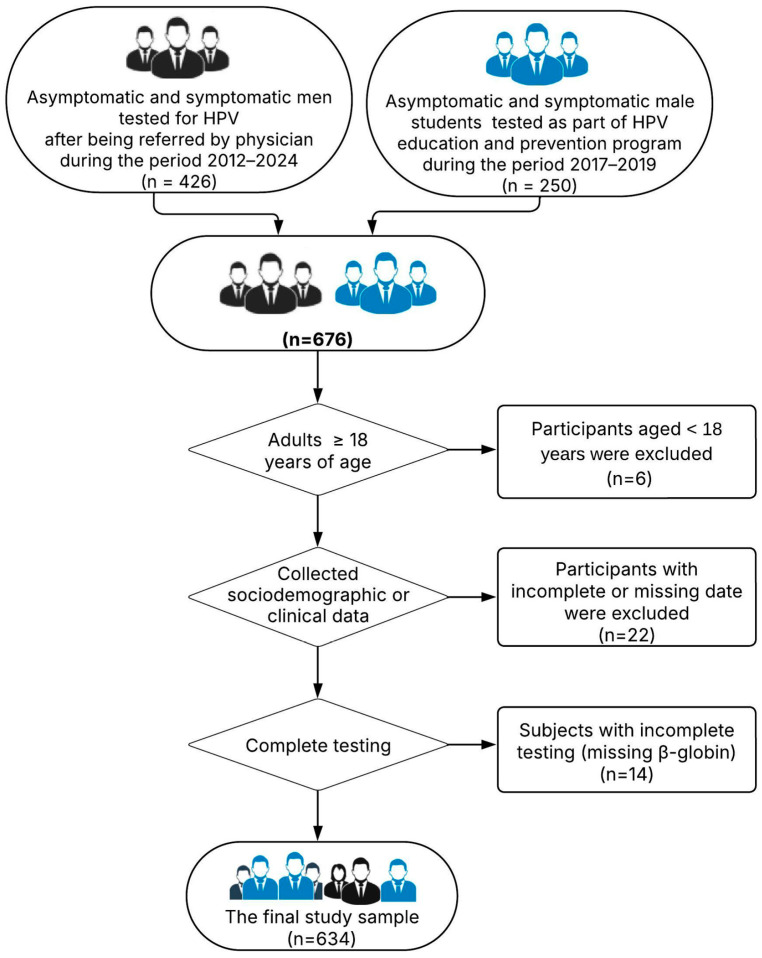
The flowchart of patients who met the inclusion/exclusion criteria for this study.

Inclusion criteria for the regularly referred patients were as follows: male individuals aged 18 years or older; the presence of a medical diagnosis and/or referral from a healthcare provider; and simultaneous testing for both high-risk (HR) and low-risk (LR) HPV types. Inclusion criteria for the voluntary project participants were as follows: male students aged 18 years or older from the University of Novi Sad; and provision of informed consent for voluntary testing and data use. Exclusion criteria, applied to both groups, included the following: age below 18 years; incomplete or missing sociodemographic or clinical data; testing performed solely for LR-HPV or solely for HR-HPV types; and insufficient biological material for analysis (e.g., β-globin-negative samples). The below flowchart shows patients who met the inclusion/exclusion criteria for this study (Figure 1).

### 2.2. Sample Collection

Prior to sample collection, all participants received specific instructions, including refraining from genital washing the day before the examination, maintaining at least one day of sexual abstinence, and avoiding urination for 1.5–2 h before the procedure. For both participant groups, swabs were collected by trained technicians from the IPHV. The urogenital swabs analyzed in this study were collected from multiple anatomical sites, including the distal urethra and external genitalia (coronal sulcus and glans penis). Regardless of the presence of genital warts or other lesions, samples were always collected from these sites. After collection, swabs were placed in tubes containing 2 mL of virus transport medium (Sigma Virocult, Medical Wire & Equipment, Corsham, UK) and stored at 2–8 °C for up to 72 h. However, if longer storage was required, samples were frozen at −80 °C. The number of swab samples corresponded to the number of participants recruited during the study periods.

### 2.3. HPV Detection and Genotyping

Viral DNA was extracted using either the DNA–sorb–A kit for manual nucleic acid extraction based on sorbent technology or the SaMag STD DNA Extraction Kit (Sacace Biotechnologies, Como, Italy), which utilizes magnetic particles for automated extraction. In the latter case, isolation was performed using the automated nucleic acid isolation station SaMag–12 Automatic System (Sacace Biotechnologies, Como, Italy). Both extraction methods were conducted according to the manufacturer’s instructions. The isolated DNA was stored at −80 °C until it was used for HPV DNA genotyping.

HPV DNA analysis was performed using real-time PCR with the following commercial kits: HPV Genotypes 14 Real-TM Quant and HPV 6/11 Real-TM (Sacace Biotechnologies, Como, Italy), as well as AmpliSens HPV HCR genotype-titre-FRT PCR kit and AmpliSens HPV 6/11–FRT PCR kit (Ecoli Dx, Prague, Czech Republic). These tests are commercially available and approved for in vitro molecular diagnostics. They are designed for the specific genotyping and quantification of 14 high-risk HPV types (HPV 16, 18, 31, 33, 35, 39, 45, 51, 52, 56, 58, 59, 66, and 68) and two low-risk HPV types (HPV 6 and 11). During the real-time PCR analyses with both commercial kits, positive and negative HPV controls were included in each run. Additionally, both kits incorporate the detection of β-globin, an internal endogenous control that verifies the presence of sufficient cellular material in the sample and the absence of inhibitors. If the amplification signal of the internal control was absent or below the set threshold, the sample was considered invalid and required reanalysis.

### 2.4. Ethical Aspect

This study was conducted in accordance with the Declaration of Helsinki and was approved by the Ethics Committee of the IPHV. The Ethics Committee approved the project “*I Have Insight into My HPV Profile*” (No. 01-1692/2, dated 8 October 2013). Written informed consent was obtained from all participants enrolled in the project. For patients undergoing routine diagnostic procedures, written patient consent was deemed unnecessary because each urogenital sample form was accompanied by a laboratory service request, which had to be signed and approved by the urologist or dermatologist responsible for obtaining verbal patient consent for each urogenital specimen collected for HPV diagnosis. This study was approved by the Ethics Committee of the Institute of Public Health of Vojvodina (No. 01-426/2, dated 11 March 2024).

### 2.5. Statistical Analyses

Descriptive statistics with absolute frequencies and percentages (%) were used to present categorical variables, and the Chi-square test (or Fisher’s exact test, where appropriate) was used to test differences in distributions between different groups, with Bonferroni multiple testing correction also being used where appropriate. We classified samples with the presence of only HPV 6 and/or HPV 11 as low-risk HPV (LR-HPV)-positive samples, and samples with the presence of at least one of the following genotypes—HPV 16, HPV 18, HPV 31, HPV 33, HPV 35, HPV 39, HPV 45, HPV 51, HPV 52, HPV 56, HPV 58, HPV 59, HPV 66 and HPV 68—as high-risk HPV (HR-HPV)-positive samples. In this study, “infection” refers to the detection of HPV DNA by real-time PCR. This detection confirms the presence of viral DNA in the sample but does not necessarily indicate active infection or clinical disease. Our study did not include a direct evaluation of Gardasil 9-induced immunity. Instead, we compared the HPV genotypes detected from samples with the genotypes included in the available vaccines, i.e., Cervarix (HPV 16 and HPV 18), Gardasil 4 (HPV 6, HPV 11, HPV 16, HPV 18), and Gardasil 9 (HPV 6, HPV 11, HPV 16, HPV 18, HPV 31, HPV 33, HPV 45, HPV 52, HPV 58). HPV-positive participants were then classified as having either completely different genotypes from those that the vaccines contain (i.e., vaccine would not have provided protection since these genotypes are not present in the vaccine), partially covered genotypes (vaccines contain some of the detected genotypes), or fully covered HPV genotypes in their sample. Additionally, we used a logistic regression model to explore the role of age as a predictive factor associated with the presence of an HPV-positive result, the presence of multiple HPV genotypes, and the presence of HR-HPV genotypes. The odds ratio (OR) with the corresponding 95% CI was calculated to demonstrate the strength of this association. Results at a *p*-value < 0.05 were considered statistically significant. All statistical analyses were performed using Stata v.17 (STATA StataCorp, College Station, TX, USA).

## 3. Results

### 3.1. Overall HPV Distribution in the Analysed Samples

**Figure 2:**
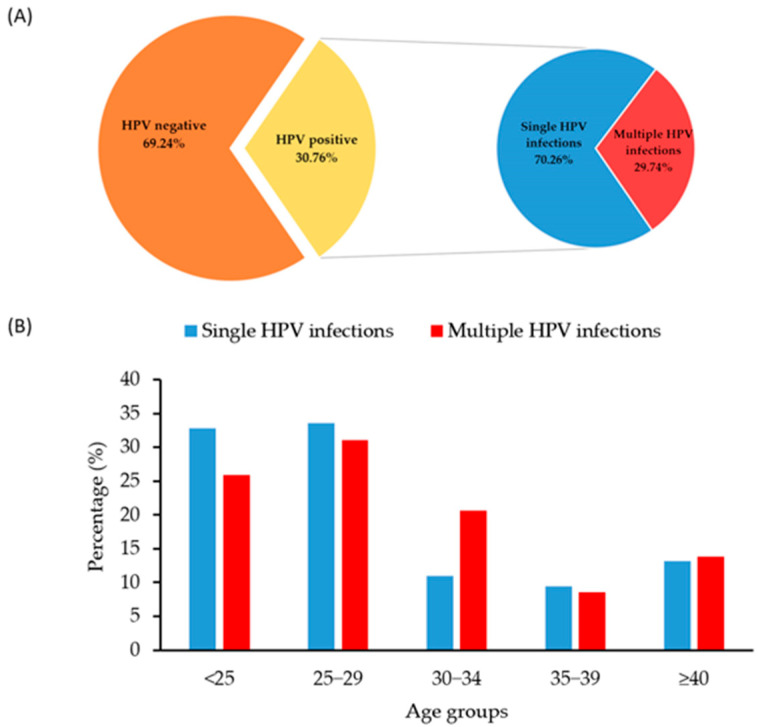
Overall HPV distribution with the ratio of HPV-positive and -negative samples (**A**); distribution of samples with detected single and multiple HPV genotypes by participants’ age category (**B**).

A total of 634 samples from men in the Autonomous Province of Vojvodina, Serbia, with a mean age of 30 years (SD = 9.98; range: 18–79 years), were analyzed for the presence of HPV between 2012 and 2024. The results indicated that 195 men tested positive for HPV, representing an overall frequency of 30.76% within the study population. The highest proportion of samples came from men under 25 years of age (35.96%), followed by those aged 25–29 (27.29%) and 30–34 (14.20%). The majority of the positive samples (70.26%) contained a single HPV genotype, while 29.74% had different combinations of multiple HPV genotypes (Figure 2A). For both types of HPV infection, the highest percentage of men was in the 25–29 age category, which accounted for 33.58% of the single HPV group and 31.03% of the multiple HPV group (Figure 2B). There was no significant difference in the distribution of single and multiple HPV-positive samples across the age categories (*p* = 0.468).

### 3.2. Genotype–Specific Distribution of Detected HPVs

**Figure 3:**
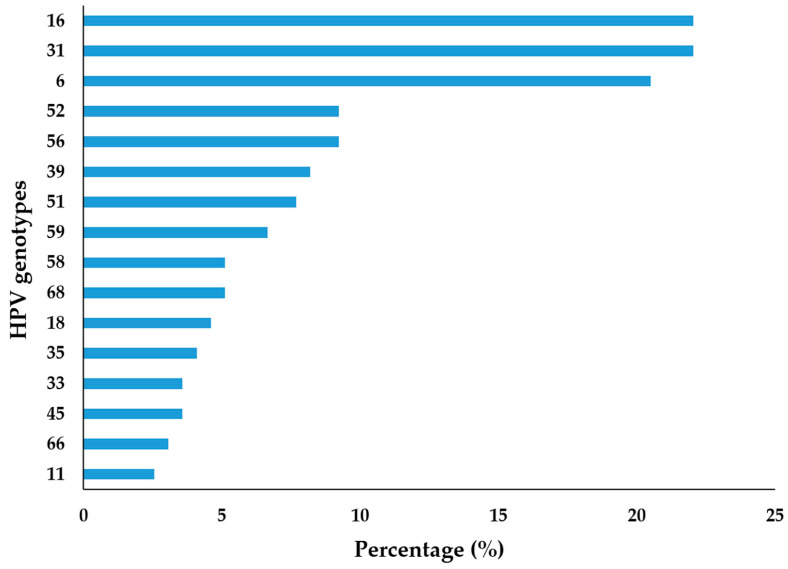
Distribution of urogenital HPV infection by genotype among the tested men in Vojvodina, Serbia.

The genotype-specific HPV distribution of the positive samples showed that HPV 16 and HPV 31 were the most frequently detected genotypes in urogenital swabs from men in our region, with both being identified in 22.05% of cases. Additionally, HPV 6 was detected in 20.51% of cases, followed by HPV 52 in 9.23%, HPV 56 in 9.23%, and HPV 39 in 8.21% (Figure 3).

### 3.3. Age–Specific Distribution of HPV

The highest HPV percentage was observed in men aged 25–29 (32.82%), followed by males under 25 (30.77%). However, there was no statistically significant difference in the distribution of HPV-positive and HPV-negative samples from individuals across the age groups (*p* = 0.256) (Table 1).

In contrast, significant differences were observed in the presence of HR-HPV and LR-HPV infections by age category (*p* < 0.001). Of the 195 HPV-positive samples, 165 (84.62%) contained at least one HR-HPV genotype, while 30 samples (15.38%) had only LR-HPV genotypes (Table 1). The highest percentages of both LR- and HR-HPV genotypes were detected predominantly in younger men. Specifically, LR-HPV genotypes were most frequently detected in younger individuals (63.33% in those <25 years old and 23.33% in 25–29-year-olds), a distribution that was significantly different from that of the HR-HPV genotypes (34.55% in 25–29-year-olds and 24.85% in those <25 years old) (*p* < 0.001).

The associations between specific HPV genotypes and age were analyzed by dividing the participants into two groups based on the previous results shown in Figure 2B: men aged 18–30 and those older than 30 years. A statistically significant difference in prevalence between these two age groups was observed only for HPV 6 (*p* < 0.001), which was significantly more common in younger men (28.91%) compared to older men (4.48%). Even though the difference was not statistically significant, HPV 31 showed a slightly higher percentage in men older than 30 years (32.84% vs. 16.41%), as did HPV 59 and HPV 68 (11.94% vs. 3.91% and 11.94% vs. 1.56%, respectively). The differences in the distribution of other HR-HPV genotypes across the two age categories were not statistically significant (*p* > 0.05), (Table 2).

### 3.4. HPV Genotype Distribution According to Clinical Manifestation

**Figure 4:**
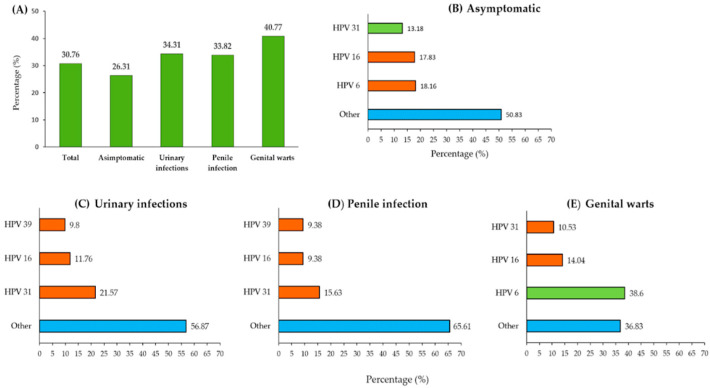
Frequency of HPV infection by diagnostic category. (**A**) Proportion of individuals with HPV DNA-positive results across different clinical categories; (**B**–**E**) distribution of the most common HPV genotypes among asymptomatic men (**B**), men with urinary infections (**C**), men with penile infections (**D**), and men with genital warts (**E**). “Other”, marked in blue, represents a pooled group that includes various HR-HPV genotypes (16, 18, 31, 33, 35, 39, 45, 51, 52, 58, 59, 66, or 68) and LR-HPV genotypes 6 or 11.

Figure 4 presents the overall frequency of HPV DNA-positive samples and the most common HPV genotypes among men with different referral diagnoses. Panel A illustrates the proportion of individuals with detected HPV DNA across the diagnostic categories, while Panels B–E depict the distribution of the most common HPV genotypes within each subgroup. Detailed percentages for each HPV genotype across the study groups are provided in Supplementary Table S1.

Asymptomatic men constituted the largest group in this study (361 out of 634 participants, with an average age of 27 years), showing an overall infection prevalence of 26.31% in our study population. The distribution of circulating HPV genotypes among the asymptomatic men revealed that HPV 16 (18.16%) was the most prevalent, followed by HPV 31 (17.83%) and HPV 6 (13.18%). Notably, these three HPV genotypes accounted for nearly half of the infections (49.17%), while the remaining 50.83% were attributed to other HPV genotypes.

The participants referred for HPV testing due to urinary tract infections (102 out of 634) were the oldest, with a median age of 38 years. They showed a prevalence of 34.31% and were most frequently positive for HPV 31 (21.57%), followed by HPV 16 (11.76%) and HPV 39 (9.8%). Other HPV genotypes collectively accounted for 56.87% of the infections in this subgroup.

Among the men diagnosed with penile infection (68 out of 634; median age: 32 years), the prevalence was 33.82%. HPV 16 was the most detected genotype (15.63%), followed by HPV 31 and HPV 18, with each being detected in 9.38% of cases. Notably, HPV 18 was relatively uncommon in the overall sample (Figure 3) but occurred more frequently in this clinical subgroup. Other HPV genotypes, aside from these three, were identified in 65.61% of cases.

The study participants who were diagnosed with genital warts (103 out of 634; mean age: 27 years) had the highest prevalence at 40.77%. LR-HPV 6 was the most commonly detected genotype (38.6%), followed by HPV 16 (14.04%) and HPV 31 (10.53%). These three genotypes accounted for 63.17% of the infections, while the remaining 36.83% were attributed to other HPV genotypes.

To examine the distribution of asymptomatic and symptomatic HPV infections across different age groups, further analysis was performed on 195 HPV-positive urogenital swab samples, as shown in Table 3.

A statistically significant difference was identified concerning the presence of symptoms and the age of the patients (χ^2^ test; χ^2^ = 14.71; *p* = 0.0053). The frequency of asymptomatic HPV infection was highest among the youngest tested patients (60%) and decreased with age. Contrarily, the incidence of symptomatic HPV infection rose with the age of the male patients, being most prevalent among patients over 40 years (76.92%).

### 3.5. The Frequency of HPV Genotypes Covered by Some Licensed Prophylactic Vaccines

**Figure 5:**
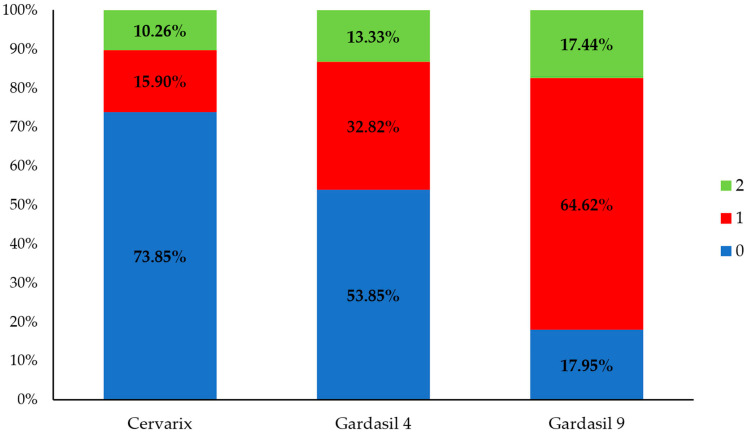
Distribution of participants with HPV DNA-positive results categorized according to the compatibility of their detected HPV genotypes with vaccine-covered genotypes. Category 0 (blue): individuals whose detected HPV genotypes are not included in the vaccine, indicating that the vaccine would not have provided protection. Category 1 (red): individuals whose detected HPV genotypes are exclusively covered by the vaccine, indicating that full protection would have been possible. Category 2 (green): individuals infected with both vaccine-covered and non-covered HPV genotypes, indicating that the vaccine would have provided only partial protection.

Subsequently, we focused on individual men, investigating the percentage of potential vaccine coverage, assuming that each person received one of the three available vaccines (Figure 5). Approximately 15.90% of individuals who hypothetically received Cervarix would be fully protected (category 1), while 10.26% would receive partial protection (category 2). In 73.85% of individuals, the vaccine would not be effective (category 0). Genotypes covered by quadrivalent Gardasil 4 were found in 46% of HPV-positive men, i.e., only vaccine-containing genotypes were seen in 32.82% of the positive samples (category 1) and those containing vaccine genotypes in combination with other genotypes were seen in 13.33% (category 2) of the positive samples. Finally, genotypes included in the nonavalent Gardasil 9 vaccine were present in 64.62% of the positive samples, while these in combination with other genotypes were present in 17.44% of the positive samples, covering a total of 82% of the men with a positive HPV finding. Specifically, only 17.95% of men fell into category 0, indicating that Gardasil 9 would be ineffective for the smallest percentage of cases.

### 3.6. Association Between Age and HPV Positive Result

We further investigated the role of age as a potential predictor of HPV positivity in men and found that those aged 25–29 years old were significantly more likely to test positive for any HPV genotype compared to those under 25 years (OR = 1.64, 95% CI: 1.07–2.52; *p* = 0.022) (Table 4). At the same time, men in the same age group were more likely to test positive for HR-HPV (OR = 2.24, 95% CI: 1.41–3.56; *p* = 0.001). Regarding the odds of acquiring HR-HPV infection, men aged 30–34 years were 1.85 times more likely (OR = 1.85, 95% CI: 1.05–3.27; *p* = 0.033) to have it compared to men from the youngest age group.

## 4. Discussion

To our knowledge, this is the first study to examine the HPV distribution among the male population from Serbia. Previous studies on HPV occurrence in our country have primarily focused on women due to its association with cervical cancer. Therefore, this topic is of great importance because understanding the true distribution of HPV in men is crucial to efforts to prevent HPV-related diseases. The analysis of urogenital swabs from 634 men revealed an overall HPV infection presence in 30.76% of the tested population. Although specific estimates may vary by region and population, the findings of our study are consistent with the global trends described in a recent systematic review. Globally, the prevalence of genital HPV infection is estimated to be 31% (95% CI: 27–35) for any HPV and 21% (95% CI: 18–24) for high–risk HPV, based on data from 65 studies conducted between 1995 and 2022 [2]. Our observed frequency (30.76%) is similar to that reported in the neighboring country of Croatia [19], while some European countries, such as the Czech Republic [20] and Slovenia [21], have slightly higher frequencies of 37% and 41%, respectively.

Estimates of HPV prevalence rates for men vary widely in the literature, ranging from 1% to 75% [2]. One of the primary reasons for this broad range is the variability in sampling strategies, particularly the selection of different anatomical sites for specimen collection. This was demonstrated in a study by Giuliano et al. which examined the HPV-DNA presence in multiple locations within the genital tract of 463 healthy men. The highest HPV detection rates were observed on the shaft of the penis (49.9%), followed by the glans penis/coronal sulcus (35.8%) and scrotum (34.2%), with lower detection rates being observed in the urethra (10.1%) [22]. Similarly, Nicolau et al. assessed the HPV-DNA frequency among 50 male partners of HPV-infected women, reporting detection rates of 44% in the internal prepuce, 30% in the distal urethra, 24% in the glans, 24% in the external prepuce of the penile shaft, 12% in the scrotum, and 8% in the anus [23]. In our study, urogenital swabs were collected from multiple anatomical sites, specifically the distal urethra and external genitalia (coronal sulcus and glans penis). Given the well-documented variations in HPV prevalence based on the choice of anatomical site for sampling, our findings should be interpreted in this context. Evidence from previous studies, including our own, underscores the need for more comprehensive research to establish a scientific consensus regarding the selection of anatomical sampling sites that are painless and acceptable to men, while providing reliable estimates of HPV occurrence.

No significant relationship was observed between age and overall HPV presence in men. Consistent with global trends, the HPV frequency peaked in the 25–29 age group, while remaining at similar levels in older age groups. The profile of HPV infection among men differs from that observed in women from our region, where women under 31 years of age had the highest percentage of positive HPV samples (55.2%) and a second peak was observed in women over 60 years of age (44.7%) [24]. These observations suggest that men are susceptible to HPV infection throughout their lives, positioning them as a permanent reservoir for transmissible HPV infections [25]. However, a separate analysis of HR-HPV and LR-HPV types indicated that their presence decreased with increasing age, with LR-HPV not being detected in men older than 40 years. These findings highlight that young adult men, especially those in their late 20s and early 30s, are at the highest risk of HR-HPV infection, emphasizing the need for targeted prevention strategies. The observed decline in HPV presence in older age groups may reflect changes in sexual behavior, immunity, or spontaneous clearance of the virus over time [25].

In addition, this investigation also aimed to assess the frequency of specific HPV types circulating among the male population in this region and to determine whether certain types are more prevalent in different age groups. The results indicated that oncogenic HPV types 16 and 31 were equally frequent, each accounting for 22.05% of the study population, followed by HPV 52 and HPV 56. In contrast, oncogenic HPV type 18 was detected at a low percentage of 4.61%, which is partially consistent with the predominant HPV types observed among women in our region. Notably, the data revealed the same frequency of HPV 16 and HPV 31 among the men in this study. HPV 16 is typically the most common genotype worldwide due to its high transmission efficiency and oncogenic potential. However, HPV 31, also a part of the Alpha-9 species, shares similar transmission routes and tissue tropism. The direct reduction of the prevalence of HPV 16 due to vaccination, variations in sexual behavior, partner networks, or cultural practices may balance the frequencies of these genotypes among men [26,27]. Random variation could also contribute to equal detection rates; therefore, this finding requires confirmation in a larger study. Consistent with observations among women in this region [24], the majority of HPV-positive men were infected with a single HPV genotype (70.26%), while 29.74% had different combinations of multiple HPV genotypes. Although no significant difference was found in the distribution of single and multiple HPV-positive samples by age category, it is notable that a slightly higher percentage of men aged 30–34 had multiple infections (10.95% vs. 20.69%). This outcome may have several explanations, including the persistence of previous HPV infections, as suggested by a prospective follow-up study among Danish soldiers [28].

HPV infections are often asymptomatic, allowing the virus to circulate silently within the population [29]. In the present study, we identified a diverse spectrum of oncogenic HPV types among asymptomatic carriers, with HPV 16 being the most dominant. High-risk HPV types, such as HPV 16 and HPV 18, are strongly linked to cervical cancer and other anogenital and oropharyngeal cancers in women, posing a continuous risk of transmission to their partners. Further analysis of 195 HPV-positive men revealed a significant association between age and symptom presentation (χ^2^ = 14.71; *p* = 0.0053); asymptomatic infections were most frequent among the youngest group (60%) and declined with age, while symptomatic infections were most common in men over 40 (76.92%). This pattern suggests that younger men often act as unknowing carriers, while clinical manifestations tend to appear later in life. These findings reinforce the need for early preventive strategies, especially HPV vaccination in adolescence, before sexual debut and potential exposure. The early identification of men’s HPV status also enables them to adopt preventive measures such as limiting their sexual partners, timely screening, smoking cessation, and considering circumcision [14].

The reasons for referring men for HPV testing are not uniform and may vary depending on the country and the approach of the treating physician. We attempted to group the underlying diagnoses for which men were referred to the IPHV for HPV testing into three categories: genital warts, penile inflammation, and urinary tract diseases.

Genital warts are the most common condition associated with HPV infection in men [29]. Although condylomas are not linked to mortality, they cause significant emotional distress and a reduced quality of life, in addition to imposing a considerable financial burden on healthcare systems [30]. In our study, approximately 103 men reported genital warts, and there was a high frequency of HPV infection in this subgroup (40.77%). Other studies have also noted that genital warts affect younger adult men, particularly those aged 25 to 29 years. However, a recent study from China reported that the highest incidence rate of genital warts was observed in the 40–44-year-old age group [31]. Although the literature indicates that 90% of condylomas are associated with low-risk HPV types 6 and 11, in our region, HPV 6 was the dominant type, while HPV 11 was rarely detected. Additionally, among individuals with genital warts, coinfection with HR-HPV was frequently detected, with HPV 16, 31, 56, and 45 being present at frequencies of 14.04%, 10.5%, 7.02%, and 3.51%, respectively. The presence of genital warts being associated with both low-risk and high-risk HPV types underscore the need for careful clinical management and counseling to reduce the risk of transmitting the infection to partners.

Approximately 11% (68/634) of the subjects in our study were referred for HPV typing due to inflammatory or irritative processes of the penis. According to published data, these inflammations are common, especially in uncircumcised men [32]. These conditions often manifest as itching, tenderness, and pain, cause significant morbidity, are difficult to treat, and increase the burden of healthcare costs. In this context, it is important to note that circumcision is not a common cultural practice among men living in the northern part of Serbia. We found a frequency of 33.82% in this group of men. A comparison of our results with those of other similar studies is difficult because only a few studies have examined the distribution of HPV in a similar cohort. In one study, HPV was detected in 56% of patient samples (of which 54% were oncogenic types), while another study detected HPV 6 in four out of five cases [33]. Among men who had penile inflammation or irritation, we detected HPV 16, HPV 18, and HPV 31 most frequently. Interestingly, HPV 18 was rarely identified overall but was found with a frequency that was immediately below that of HPV 16 in the group with penile inflammation. HPV 16 and HPV 18 are known to be associated with malignant genital diseases, with HPV 16 being implicated in approximately 31% of penile cancers, making it the dominant subtype [34].

In our study, a subgroup of approximately 102 men was referred by urologists for HPV testing due to various urinary tract conditions. Among these individuals, 34.31% tested positive for HPV. Notably, HPV 31 was detected twice as often compared to HPV 16 (21.57% vs. 11.76%). The anatomical proximity of the genital and urinary tracts may increase the risk of HPV infection in the urinary tract. Consequently, HPV is often investigated as a potential etiological agent in the development of urinary tract cancers. Some studies suggest that HR-HPV may contribute to the progression from benign prostatic conditions to malignancy by promoting cellular immortalization; however, a direct causal relationship has not yet been established [35]. Moreover, although a relatively high prevalence of this virus has been observed in individuals with bladder cancer, a meta-analysis of 26 relevant articles found no convincing evidence that supported a role of HPV in the development of this type of cancer [36]. Our findings contribute to the global data on HPV prevalence in male urinary tract conditions, emphasizing the need for further research to determine the potential role of specific HPV genotypes in urological diseases.

In light of the substantial burden of HPV-related diseases among men, recent global health initiatives have been undertaken to advocate for the adoption of gender-neutral vaccination programs. A report by Baker and Winterflood (2024) emphasizes that approximately one in five men worldwide carries a high-risk HPV infection, which contributes to an estimated 180,000 new cancer cases annually in men [37]. These figures underscore the necessity of even further including boys in vaccination efforts to effectively reduce the incidence of HPV-related cancers in both sexes. It is also crucial to acknowledge the significant role that men play in transmitting HPV to women [37]. The transmission of HPV between sexual partners often leads to persistent infections, thereby increasing the risk of high-grade cervical lesions and cervical carcinogenesis [14]. As persistent infections with high-risk oncogenic HPV genotypes are the leading cause of cervical cancer [38], male sexual behavior is a critical factor in the development of this disease in women [14]. Furthermore, evidence suggests that female-only HPV vaccination strategies may not provide sufficient protection for unvaccinated individuals due to the high prevalence of oncogenic HPV genotypes in the unvaccinated population [39]. In contrast, male vaccination is expected to provide indirect benefits for females through the enhancement of herd immunity, a reduction in the viral load within the general population, and a decreased incidence of anogenital diseases in males [40]. This approach is particularly pertinent for countries like Serbia, where our study in men identified a significant frequency of HPV genotypes that are targeted by the Gardasil-9 vaccine. Expanding the HPV vaccination coverage among boys in Serbia can enhance herd immunity, thereby providing comprehensive protection against HPV-related diseases for all individuals.

Although our study provides valuable insights into the frequency and distribution of certain HR and LR-HPV types in northern Serbia, several limitations must be acknowledged. First, the retrospective design of this study and the lack of detailed data on sexual behavior, sexual orientation, and HPV vaccination status limited our ability to assess specific risk factors. Second, this study did not include patients from the entire territory of Serbia but only from its northern part, the Autonomous Province of Vojvodina. Therefore, this study is not population-based and does not represent the general male population in Serbia. Third, the participants who agreed to HPV testing might differ from those who did not, which could potentially introduce self-selection bias and may limit the generalizability of the results. However, it is important to highlight that this study is the first to investigate HPV distribution among men in Serbia and, thus, it fills a significant gap in the regional epidemiological data. Additionally, conducting diagnostic analyses in a single, centralized laboratory minimized inter-laboratory variability, thereby enhancing the consistency and reliability of the results.

## 5. Conclusions

Our study revealed a high occurrence of genital HPV infection among men in northern Serbia. This finding underscores the importance of comprehensive HPV prevention and control measures for the male population, contributing to ongoing research efforts aimed at reducing the burden of HPV-associated diseases in our region. Vaccinating men against HPV enhances herd immunity, providing additional protection to their female partners. Additionally, given the current lack of a gold standard for HPV testing in men, optimizing sampling techniques and HPV detection methods is crucial for enhancing disease monitoring and prevention strategies. A significant recent increase in certain HPV-related cancers affecting men further emphasizes the importance of HPV vaccination as a preventive measure. Therefore, to achieve these public health objectives and effectively mitigate the risk of HPV transmission and associated diseases in all individuals, regardless of sex or sexual orientation, it is essential to prioritize educational initiatives and expand HPV vaccination programs to include males.

## Figures and Tables

**Table 1:** Distribution of the HPV testing results based on the participant’s age.

Age Group	HPV (Any) Positive, n (%)	HPV Negative, n (%)	Total, n (%)	*p*-Value *	HR–HPV, n (%)	(Only) LR–HPV, n (%)	Total, n (%)	*p*-Value *
<25	60 (30.77)	168 (38.27)	228 (35.96)	0.256	41 (24.85)	19 (63.33)	60 (30.77)	**<0.001**
25–29	64 (32.82)	109 (24.83)	173 (27.29)		57 (34.55)	7 (23.33)	64 (32.82)	
30–34	27 (13.85)	63 (14.35)	90 (14.20)		26 (15.76)	1 (3.33)	27 (13.85)	
35–39	18 (9.23)	42 (9.57)	60 (9.46)		15 (9.09)	3 (10.00)	18 (9.23)	
≥40	26 (13.33)	57 (12.98)	83 (13.09)		26 (15.76)	0 (0)	26 (13.33)	
total	195 (100)	439 (100)	634 (100)		165 (100)	30 (100)	195 (100)	

* Using Chi–square or Fisher’s exact test. In the bold is the statistically significant result at *p* < 0.05.

**Table 2:** Age-specific distribution of male participants with different HPV genotypes.

HPV Genotype	Total, n (%)	Age Category, n (%)		*p*-Value *
		18–30 Years	>30 Years	
HPV 6	40 (20.51)	37 (28.91)	3 (4.48)	**<0.001**
HPV 11	5 (2.56)	4 (3.13)	1 (1.49)	0.662
HPV 16	43 (22.05)	28 (21.88)	15 (22.39)	0.935
HPV 18	9 (4.62)	4 (3.13)	5 (7.46)	0.279
HPV 31	43 (22.05)	21 (16.41)	22 (32.84)	0.009
HPV 33	7 (3.59)	6 (4.69)	1 (1.49)	0.425
HPV 35	8 (4.10)	6 (4.69)	2 (2.99)	0.717
HPV 39	16 (8.21)	7 (5.47)	9 (13.43)	0.054
HPV 45	8 (4.10)	5 (3.91)	3 (4.48)	0.999
HPV 51	15 (7.69)	11 (8.59)	4 (5.97)	0.514
HPV 52	18 (9.23)	12 (9.38)	6 (8.96)	0.923
HPV 56	18 (9.23)	12 (9.38)	6 (8.96)	0.923
HPV 58	10 (5.13)	6 (4.69)	4 (5.97)	0.739
HPV 59	13 (6.67)	5 (3.91)	8 (11.94)	0.065
HPV 66	6 (3.08)	3 (2.34)	3 (4.48)	0.415
HPV 68	10 (5.13)	2 (1.56)	8 (11.94)	0.003

* Differences in HPV genotype frequency between the two age groups were assessed using the Chi-square test, with Fisher’s exact test applied where appropriate. Bonferroni correction was applied to account for multiple comparisons, adjusting the significance threshold to *p* < 0.0016 (0.05/32). Statistically significant results after the correction are presented in bold.

**Table 3:** Age-specific distribution of male patients with different diagnostic categories.

Diagnostic Category	Age Category (Years), n (%)					Total n (%)	χ^2^	*p*-Value
	<25	25–29	30–34	35–39	≥40			
Asymptomatic	36 (60.00)	36 (56.25)	12 (44.44)	5 (27.78)	6 (23.08)	95 (48.72)	14.71	0.0053 **
Symptomatic	24 (40.00)	28 (43.75)	15 (55.56)	13 (72.22)	20 (76.92)	100 (51.28)		
Total	60 (30.77)	64 (32.82)	27 (13.85)	18 (9.23)	26 (13.33)	195 (100)		

n–number of HPV-positive cases; ** *p* < 0.01.

**Table 4:** Logistic regression model for the association of age with the presence of HPV positive result, multiple HPV, and HR-HPV genotypes.

Variables	Any HPV		Multiple HPV		HR–HPV Tip	
	OR (95% CI)	*p*-Value	OR (95% CI)	*p*-Value	OR (95% CI)	*p*-Value
age, years	1.01 (0.99–1.03)	0.222	1.01 (0.98–1.03)	0.593	**1.02 (1.01–1.04)**	**0.01**
Age category						
<25	ref		ref		ref	
25–29	**1.64 (1.07–2.52)**	**0.022**	1.65 (0.81–3.37)	0.171	**2.24 (1.41–3.56)**	**0.001**
30–34	1.20 (0.70–2.06)	0.507	2.18 (0.98–4.87)	0.056	**1.85 (1.05–3.27)**	**0.033**
35–39	1.20 (0.64–2.24)	0.568	1.29 (0.45–3.71)	0.635	1.52 (0.77–2.99)	0.224
≥40	1.28 (0.74–2.21)	0.383	1.52 (0.62–3.72)	0.365	**2.08 (1.17–3.69)**	**0.012**

Odds ratios (OR) with 95% confidence intervals (CI) are presented for each age group, with men under 25 years serving as the reference category. OR = odds ratio; 95% CI = 95% confidence interval. Statistically significant results (*p* < 0.05) are shown in bold.

## Data Availability

The data that support the findings of this study are available from the corresponding author upon reasonable request.

## Supplementary Materials

The following supporting information can be downloaded at: https://www.mdpi.com/article/10.3390/pathogens14060558/s1, Table S1: Distribution of HPV genotypes based on clinical manifestations.

## Abbreviations

The following abbreviations are used in this manuscript:

HPV	Human papillomavirus
HR–HPV	High–risk HPV types
LR–HPV	Low–risk HPV types
IPHIV	Institute for Public Health of Vojvodina
