# Supplementary material for: Detection of Human Papillomavirus in Urogenital Swabs from Male Patients in Northern Serbia
Source: Pathogens. 2025 Jun 3;14(6):558. doi: 10.3390/pathogens14060558 (PMC12195706; doi:10.3390/pathogens14060558)
Supplement: Supplementary file 1 [file pathogens-14-00558-s001.zip › pathogens-3545252-supplementary.pdf]

Supplementary Materials

Table S1. Distribution of HPV genotypes based on clinical manifestations.

HPV genotypes	Asymptomatic	Urinary Infections	Penile Inflammation	Genital Warts
HPV 6	13.18%	1.96%	0.00%	38.60%
HPV 11	1.55%	0.00%	0.00%	5.26%
HPV 16	18.60%	11.76%	15.63%	14.04%
HPV 18	2.33%	5.88%	9.38%	0.00%
HPV 31	17.83%	21.57%	9.38%	10.53%
HPV 33	4.65%	0.00%	0.00%	1.75%
HPV 35	3.10%	1.96%	6.25%	1.75%
HPV 39	5.43%	9.80%	6.25%	3.51%
HPV 45	1.55%	5.88%	0.00%	5.26%
HPV 51	5.43%	7.84%	12.50%	0.00%
HPV 52	8.53%	3.92%	9.38%	3.51%
HPV 56	6.20%	5.88%	9.38%	7.02%
HPV 58	4.65%	1.96%	3.13%	3.51%
HPV 59	3.88%	7.84%	9.38%	1.75%
HPV 66	1.55%	5.88%	3.13%	0.00%
HPV 68	1.55%	7.84%	6.25%	3.51%
